# Recent Advances in the Stereoselective Total Synthesis of Natural Pyranones Having Long Side Chains

**DOI:** 10.3390/molecules25081905

**Published:** 2020-04-20

**Authors:** Satya Kumar Avula, Biswanath Das, Rene Csuk, Ahmed Al-Rawahi, Ahmed Al-Harrasi

**Affiliations:** 1 Natural and Medical Sciences Research Center, University of Nizwa, P.O. Box 33, Birkat Al Mauz 616, Nizwa, Oman; chemisatya@unizwa.edu.om (S.K.A.); biswanathdas@unizwa.edu.om (B.D.); chancellor@unizwa.edu.om (A.A.-R.); 2 Organic Chemistry, Martin-Luther-University Halle-Wittenberg, Kurt-Mothes-Str. 2, d-06120 Halle (Saale), Germany; rene.csuk@chemie.uni-halle.de

**Keywords:** pyranone, side chain, natural product, total synthesis, stereoselectivity

## Abstract

Pyranone natural products have attracted great attention in recent years from chemists and biologists due to their fascinating stereoisomeric structural features and impressive bioactivities. A large number of stereoselective total syntheses of these compounds have been described in the literature. The natural pyranones with long side chains have recently received significant importance in the synthetic field. In the present article, we aim to review the modern progress of the stereoselective total syntheses of these natural pyranones containing long-chain substituents.

## 1. Introduction

**Figure 1:**
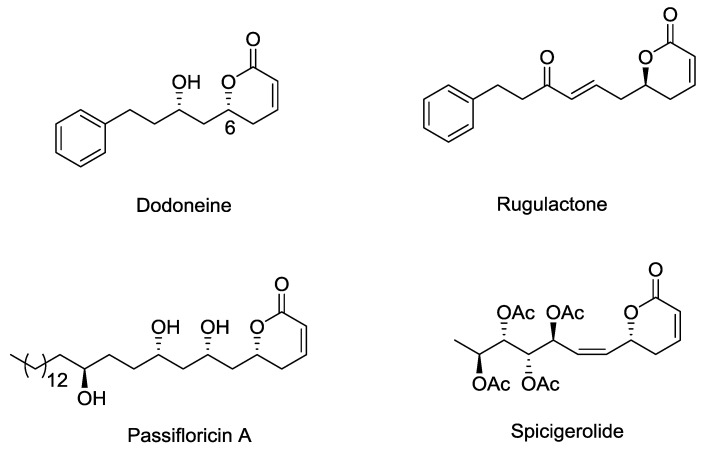
Structures of natural pyranone having long side chains.

Pyranones are an important class of natural products [1]. Several natural pyranones have been found to contain long side chains. The side chain is generally present at the C-6 portion of the pyranone ring. These naturally occurring pyranones with long-chain substituents have recently drawn considerable attention from the scientific community due to their impressive structural characteristics as well as interesting biological activities. Structurally, they contain a lactone ring, which is usually within the framework of a 5,6-dihydropyran-2-one (α,β-unsaturated δ-lactone). The H-6 in these molecules may be with the relative stereoposition of α or β. For example, in dodoneine [2] and rugulactone [3], the stereoposition of H-6 is the opposite (Figure 1).

The side chains of the pyranones may be of various lengths; they may even contain more than 20 carbons. Thus, the side chain of passifloricin A consists of 21 carbons [4]. These side chains possess several stereogenic centers with various functionalities. Generally hydroxyl, acetoxy, and carbonyl groups are located at different positions of the side chains. However, natural pyranones (such as rugulactone) having no chiral center in the side chains are also observed. Some compounds are found to possess a double bond [(*E*) or (*Z*) stereostructure] in their side chains. Rugulactone contains an (*E*)-double bond while spicigerolide has a (*Z*)-double bond in their respective side chains [3,5].

The bioactivity of this class of compounds is promising. They are found to exhibit manifold biological properties including anticancer [3,6], antiviral [7], antifungal [8], antituberculosis [9], and antimicrobial [10] activities. The α,β-unsaturated lactone moiety plays an important role in the bioactivity as it can act as a Micheal acceptor in the presence of protein functional groups [11].

The interesting structural features and important bioactivities of these molecules have inspired synthetic chemists to explore their total syntheses [12,13,14] and biologists to discover novel therapeutics [15,16,17]. In several cases, the pyranones obtained from natural sources are insufficient to conduct further experiments. The total synthesis can generate the compounds in larger amounts required to explore their new medicinal values. Total syntheses are also useful to verify the established structures of the molecules.

Various modern synthetic approaches have now been applied to construct the natural pyranone molecules with proper stereostructures. Efficient diastereoselective and enantioselective synthetic protocols have been employed to introduce the required chirality in their side chains; ring-closing metathesis (RCM) and cross-metathesis (CM) reactions [18] have frequently been applied to construct the lactone rings and side chains, respectively. Various improved approaches have currently been utilized by different synthetic chemists to accomplish successfully stereoselective syntheses of natural pyranones. In this review, we have described the recent progress of the total syntheses of these compounds having long side chains. We have focused our discussion on the total syntheses of the molecules, which have repeatedly been constructed by applying various modern synthetic protocols.

## 2. Stereoselective Total Syntheses

In recent years, different research groups utilized various efficient procedures for the stereoselective syntheses of naturally occurring pyranones having long-chain substituents. The total syntheses of the following molecules will highlight the current advances in the field.

### 2.1. Dodoneine


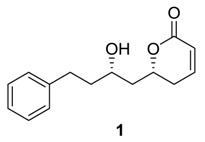


Dodoneine (**1**), a naturally occurring 5,6-dihydropyran-2-one, was isolated from a medicinal parasitic plant, *Tapinunthus dodoneifolius* that grows on the sheanut tree in West Africa [2]. The structure of the compound was derived from its spectroscopic data and X-ray crystallographic analysis of its camphorsulfonate derivative. Dodoneine (**1**) was found to possess relaxation effects on preconstricted rate aortic rings. The compound was also evaluated as a hypotensive agent and as an inhibitor of human carbonic anhydrases [19,20].

**Scheme 1:**
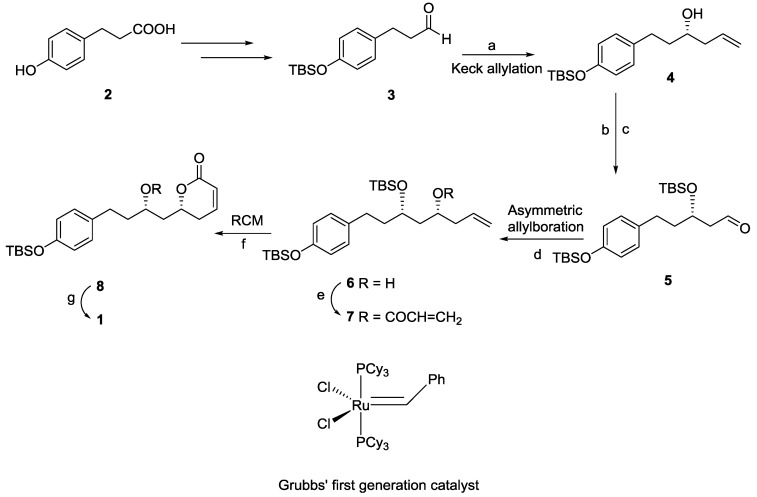
Reagents and conditions: (a) allyltri-*n*-butyltin, (*R*)-BINOL, Ti(*^i^*PrO)_4_, (approximately 60% overall); (b) TBSOTf, CH_2_Cl_2_, 2,6-lutidine, r.t., 2 h, 85%; (c) O_3_, −78 °C to r.t., PPh_3_, 2 h; (d) (+)-Ipc_2_BCl, Et_2_O,allylMgBr, −90 °C, 2 h, (approximately 60% overall); (e) CH_2_=CHCOCl, CH_2_Cl_2_, *^i^*Pr_2_Net, −78 °C, 62%; (f) 10% Grubbs first-generation catalyst, CH_2_Cl_2_, Δ, 4 h, 84%; (g) aq HF, MeCN, r.t., 16 h, 89%.

Dodoneine (**1**) has recently been synthesized by several research groups [21,22,23,24,25,26,27,28,29]. The synthetic methods generally involve the asymmetric allylation of an aldehyde for introducing the stereogenic centers and the formation of the pyranone ring by ring-closing metathesis (RCM) or intramolecular transesterification. The first total synthesis of dodoneine (**1**) was reported independently by Falomir et al. [21] and Srihari et al. [22]. Falomir et al. used commercially available dihydro *p*-coumaric acid (**2**) as the starting material (Scheme 1). The acid was converted to silylated dihydro *p*-coumaraldehyde (**3**), which underwent asymmetric Keck allylation [30] to generate the homoallylic alcohol **4** (ee ca. 95%). The silylation of **4** and ozonolysis of the product afforded aldehyde **5**. Asymmetric allylboration of **5** using (+)-Ipc_2_BCl/allylmagnesiumbromide (Ipc = isopinocamphenyl) yielded the homoallylic alcohol **6**, which was obtained as a single diastereomer after purification. The subsequent acrylation of **6** with acryloyl chloride followed by ring-closing metathesis of the acrylate (**7)** applying a Grubbs first-generation catalyst provided the pyranone **8**. Finally, the cleavage of the two silyl groups of **8** using aqueous HF in MeOH furnished dodoneine (**1**).

**Scheme 2:**
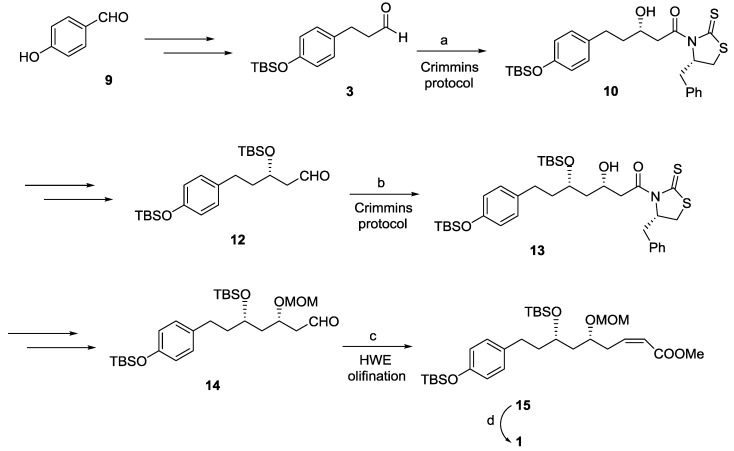
Reagents and conditions: (a) TiCl_4_, DIPEA, CH_2_Cl_2_, −78 °C, 85%; (b) TiCl_4_, DIPEA, CH_2_Cl_2_, −78 °C, 81%; (c) (CF_3_CH_2_O)_2_P(O)CH_2_COOCH_3_, NaH, THF, −78 °C, 82%; (d) PTSA or PPTS or 3 mol% HCl.

Srihari et al. applied the aldehyde **3** prepared from 4-hydrobenzaldehyde (**9**) (Scheme 2) [22]. The aldehyde **3** was reacted with (*S*)-1-(4-benzyl-2-thioxothiazolidin-3-yl) ethanone in the presence of TiCl_4_ following the Crimmins protocol [31]. The major *syn* product **10** was converted to TBS ether **11**, which upon treatment with DIBAL-H yielded the aldehyde **12**. The aldehyde **12** underwent the Crimmins aldol reaction with (*S*)-1-(4-benzyl-2-thioxothiazolidin-3-yl) ethanone and TiCl_4_ to produce the 1,3-*syn* compound **13** as the major product. The hydroxyl group of **13** was protected with MOMCl, and product was treated with DIBAL-H to form the aldehyde **14**. The latter was treated with bis-(2,2,2-trifluoromethyl) (methoxycarbonylmethyl) phosphonate following the Horner–Wadsworth–Emmons olefination reaction [32] to produce the *cis*-olefinic ester **15**. At the final stage, it was observed that 3 mol% HCl solution afforded the best result for the simultaneous deprotection and cyclization of the ester **15** to generate dodoneine **1**.

**Scheme 3:**
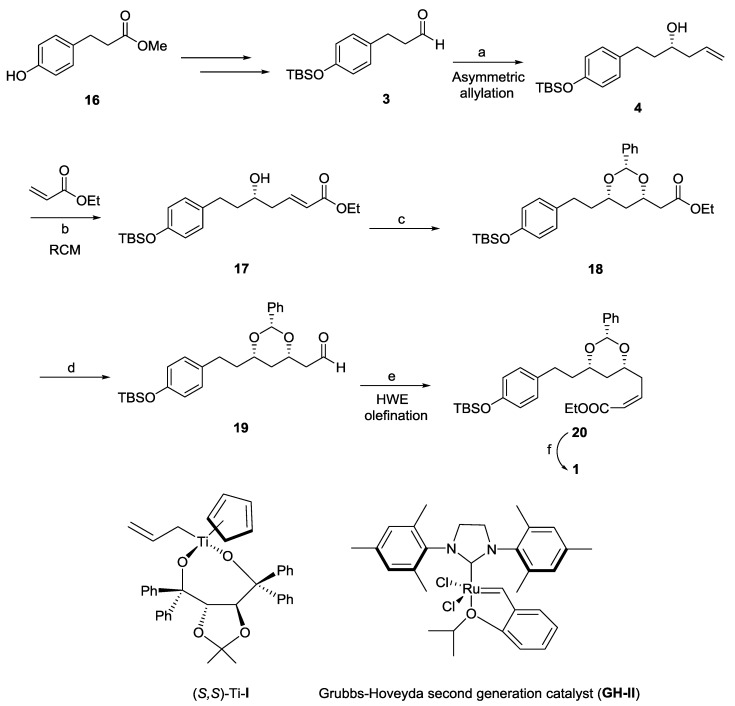
Reagents and conditions: (a) (*S,S*)-Ti-I, Et_2_O, −78 °C, 3 h, 97%; (b) GH-II (5 mol%), CH_2_Cl_2_, r.t., 2 d, 80%; (c) PhCHO, *t*-BuOK, THF, 0 °C, 2 h, 68%; (d) DIBAL-H, toluene, −78 °C, 94%; (e) (CF_3_CH_2_O)_2_P(O)CH_2_COOEt, KHMDS, 18-C-6, THF, −78 °C, 2 h, 70%; (f) aq AcOH, 60 °C, 24 h, 70%.

A short and efficient synthesis of dodoneine (**1**) was reported by Cossy et al. [23]. They prepared the aldehyde **3** from the ester **16** (Scheme 3). This aldehyde (**3**) was treated with allyl titanium complex (*S,S*)-Ti-I to produce the homoallylic alcohol **4** (*ee* 96%). A cross-metathesis reaction [33] of **4** with ethyl acrylate in the presence of a Grubbs–Hoveyda second-generation catalyst afforded the unsaturated ester **17**. On treatment with benzaldehyde using *tert*-BuOK, the ester **17** furnished the protected 1,3-diol **18** (*syn*:*anti*; 98:2). Reduction of the compound **18** with DIBAL-H afforded the aldehyde **19**, which was subjected to Horner–Wadsworth–Emmons reaction [34] using bis (2,2,2-trifluoromethyl) (methoxycarbonylmethyl) phosphonate to produce the unsaturated ester **20** (*Z*:*E* = 90:10). Finally, the treatment of **20** with 80% aq. AcOH afforded dodoneine (**1**).

**Scheme 4:**
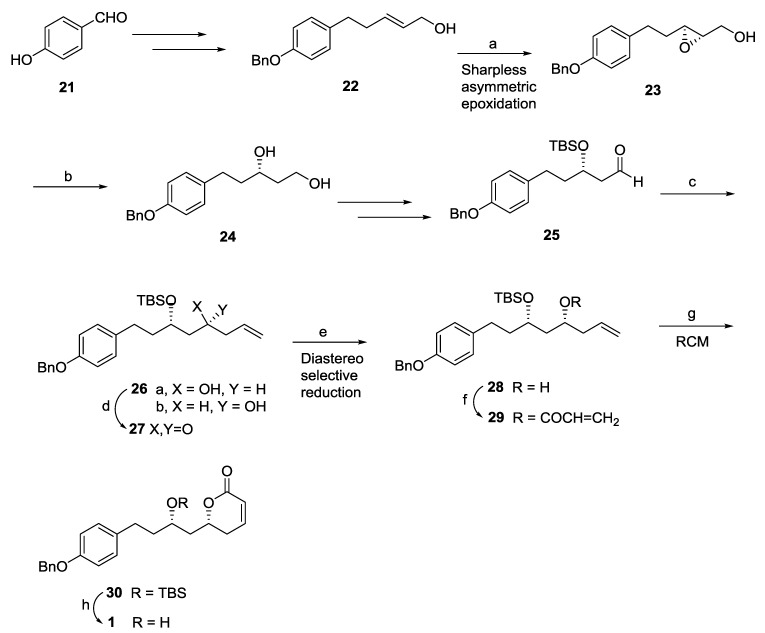
Reagents and conditions: (a) Ti(*^i^*PrO)_4_, (1.0 equiv), (+)-DIPT (1.1 equiv), TBHP (2.5 equiv), CH_2_Cl_2_, −20 °C, 12 h, 92%; (b) Red-Al (3.0 equiv), THF, 0 °C, 0.5 h, 82%; (c) CH_2_=CHCH_2_MgBr, Et_2_O, 0 °C, 1 h, 74%; (d) DMP, NaHCO_3_, CH_2_Cl_2_, 0 °C to r.t., 1 h, 88%; (e) LiAlH_4_–LiI, Et_2_O, −100 °C, 30 min, 94%; (f) acryloyl chloride, Et_3_N, 0 °C to rt, 30 min, 96%; (g). Grubbs first-generation catalyst, CH_2_Cl_2_, 50 °C, 24 h, 85%; (h) TiCl_4_, DCM, 0 °C, 89%.

Das et al. utilized 4-hydroxy benzaldehyde as the starting material and applied Sharpless asymmetric epoxidation, 1,3-*syn* diastereoselective reduction, and Grubbs ring-closing metathesis in their synthetic sequence for the stereoselective construction of dodoneine (**1**) (Scheme 4) [24].

Sharpless asymmetric epoxidation [35] of **22** was carried out with (+)-DIPT and the diastereoselective reduction of the ketone **27** with LiAlH_4_-LiI at −100 °C (*syn*:*anti* = 94:6). The intramolecular metathesis reaction of **29** was conducted using a Grubbs catalyst of the first generation.

**Scheme 5:**
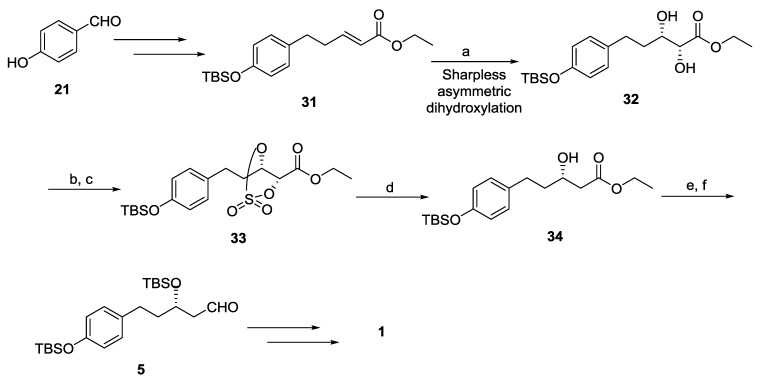
Reagents and conditions: (a) AD-mix-α, MeSO_2_NH_2_, *t*-BuOH-H_2_O (1:1), 0 °C, overnight, 85%; (b) SOCl_2_, Et_3_N, CH_2_Cl_2_, 0 °C, 30 min, 94%; (c) RuCl_3_, NaIO_4_, CCl_4_-MeCN-H_2_O: 2:2:3, 0 °C, 30 min, 92%; (d) NaBH_4_, DMF, 15 min, then THF, cat. Conc. H_2_SO_4_, cat. H_2_O, 20 min, 90%; (e) TBSCl, imidazole, CH_2_Cl_2_, 0–25 °C, overnight, 95%; (f) DIBAL-H, CH_2_Cl_2_, −78 °C, 10 min, 88%.

In another synthesis, Sharpless asymmetric dihydroxylation [36] and the regioselective nucleophile opening of cyclic sulfate formed from the resulting diol were used to generate the required chiral center (Scheme 5).

**Scheme 6:**
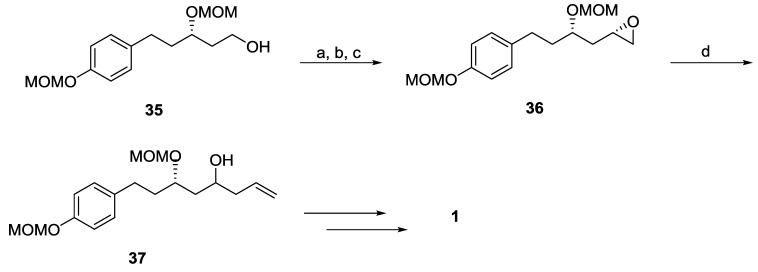
Reagents and conditions: (a) IBX/DMSO, CH_2_Cl_2_, 0 °C to r.t., 5 h; (b) trimethylsulfonium iodide, DMSO, NaH, THF, 0 °C to r.t., 6 h, 60%; (c) (*S*,*S*)-Jacobsen catalyst, AcOH, H_2_O, 12 h, 43%; (d) vinylmagnesium bromide, CuI, THF, 0 °C, 30 min, 85%.

Sabitha et al. completed the synthesis of dodoneine (**1**) starting from the known chiral alcohol **35** (Scheme 6) [26]. The latter was oxidized with IBX to the corresponding aldehyde, which was treated with trimethylsulfoxiumiodide using NaH in DMSO-THF to afford a racemic epoxide. Jacobson’s hydrolytic kinetic resolution (HKR) of this epoxide by applying (*S*,*S*)-Salen-Co-OAc catalyst yielded the chiral epoxide **36** (*ee* 95%) [37]. The epoxide **36** was converted into the homoallylic alcohol **37** by treatment with vinyl magnesium bromide and CuI. The compound is structurally related to **6**. It was subsequently transformed into dodoneine (**1**) following a similar reaction sequence as shown earlier in Scheme 1.

**Scheme 7:**
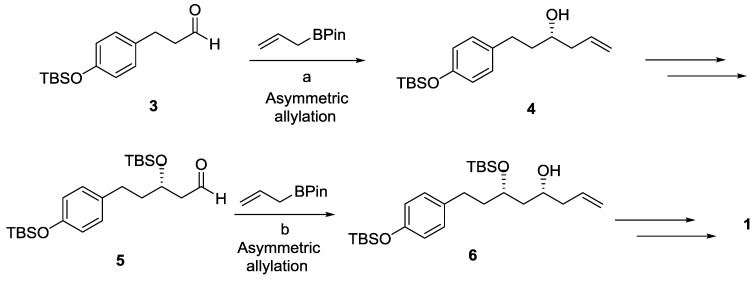
Reagents and conditions: (a) (*R*,*R*)-*p*-F-vivol **4b** (5 mol%), SnCl_4_ (3.8 mol%), Na_2_CO_3_, 4 Å MS, toluene [1.3 M], −78 °C, 4 h, 99%; (b) (*R*,*R*)-*p*-F-vivol **4b** (5 mol%), SnCl_4_ (3.8 mol%), Na_2_CO_3_, 4 Å MS, toluene [1.0 M], −78 °C, 4 h, 96%.

Rauniyar and Hall prepared the chiral alcohol **4** (*ee* 97%) from the aldehyde **3** by using *p*-F-Vivol.SnCl_4_ catalyzed allylboration with allylboron pinacolate (Scheme 7) [27]. In a similar manner, the chiral diol **6** (*dr* 99:1) was produced from the aldehyde **5**. Compound **6** was subsequently converted to dodoneine (**1**) following a sequence similar to that of Macro et al. [21] (Scheme 1).

**Scheme 8:**
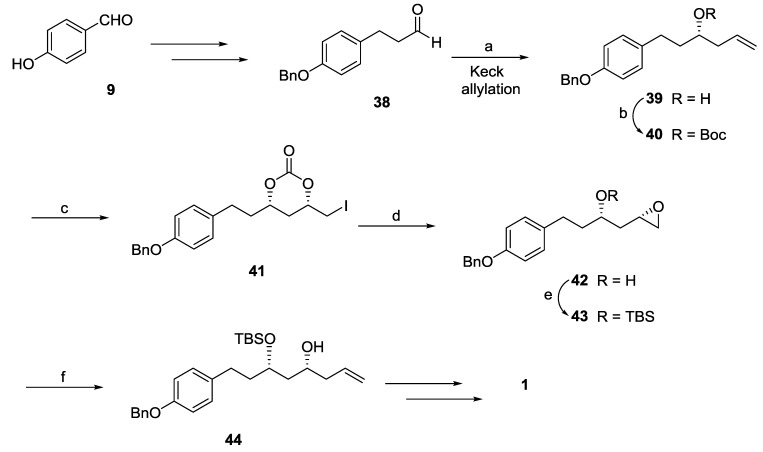
Reagents and conditions: (a) (*R*)-BINOL, 4 Å MS, Ti(*^i^*PrO)_4_, allyltributylstannane, CH_2_Cl_2_, −78 to −20 °C, 72 h, 79%; (b) (Boc)_2_O, DMAP, MeCN, r.t., 5 h, 94%; (c) I_2_, MeCN, −20 °C, 12–15 h, 82%; (d) K_2_CO_3_, MeOH, 0 °C to r.t., 2 h, 86%; (e) ^t^BuMe_2_SiCl, 1*H*-imidazole, CH_2_Cl_2_, r.t., 4.5 h, 88%; (f) CH_2_=CHMgBr, Et_2_O, 0 °C, 1.5 h, 76%.

In an alternative approach [28], the total synthesis of dodoneine (**1**) was achieved by applying Keck’s asymmetric allylation, iodine-induced electrophilic cyclization, and ring-closing metathesis (Scheme 8). Compound **40** underwent diastereoselective iodolactoxization with I_2_ to form the cyclic iodocarbonate **41** as a single diastereoisomer. This iodocarbonate (**41**) when kept in basic MeOH solution afforded *syn*-epoxy alcohol **42**. The free hydroxyl group of **42** was protected to form TBS-ether **43**, which was treated with allylmagnesiumbromide to furnish a diastereoisomeric mixture (*syn*:*anti* = 43:57). The desired *syn*-epoxy alcohol **44** was purified by column chromatography and was converted to dodoneine (**1**).

**Scheme 9:**
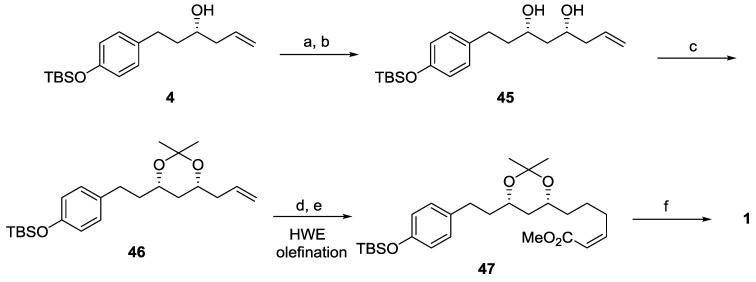
Reagents and conditions: (a) OsO_4_, 2,6-lutidine, NaIO_4_, dioxane-H_2_O (3:1), r.t.; (b) allylSiMe_3_, SnCl_4_, CH_2_Cl_2_, −78 °C, 12 h, 80%; (c) 2,3-DMP, PPTS, CH_2_Cl_2_, r.t., 2 h, 96%; (d) OsO_4_, 2,6-lutidine, NaIO_4_, dioxane-H_2_O (3:1); (e) 2-[bis(2,2,2-trifluoroethoxy)phosphoryl]acetate, KHMDS, 18-crown-6, THF, −78 °C, 4 h, 51%; (f) 80% aq AcOH, 60 °C, 1 d, 68%.

Allais and Ducrot prepared the chiral homoallylic alcohol **4 [29]** following the method developed by Rauniyar and Hall (Scheme 9) [27]. This alcohol **4** was treated with OsO_4_ and NaIO_4_ to form the corresponding β-hydroxyaldehyde, which was reacted with trimethylallylsilane and SnCl_4_ to produce the diol **45** (*dr* > 97:3) favoring the *syn*-product. The diol **45** was converted to a ketal **46**. The latter underwent an oxidative cleavage with OsO_4,_ NaIO_4_ and the resulting aldehyde was subjected to Horner–Wardsworth–Emmons olefination to furnish the unsaturated ester **47**, (Z:E = 90:10). At the final step, the treatment of **47** with 80% aq. AcOH afforded dodoneine (**1**).

### 2.2. Rugulactone


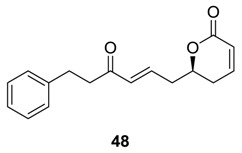


Rugulactone (**48**) was isolated from *Cryptocarya rugulosa* [3]. It contains only one chiral center at C-6 with *R*-stereoconfiguration and an α, β-unsaturated γ-lactone along with an α, β-unsaturated ketone. The compound was found to inhibit constitutive NF-kB activity in human lymphoma cell lines. Several syntheses of rugulactone (**48**) have recently been reported [38,39,40,41,42,43,44,45,46,47,48]. In these syntheses, the chirality has been introduced by applying different methodologies such as Jacobsen’s hydrolytic kinetic resolution of epoxides, Keck/Maruoka asymmetric allylation, chemoenzymatic process, the chiral pool approach, and allylation with chiral boronic esters.

**Scheme 10:**
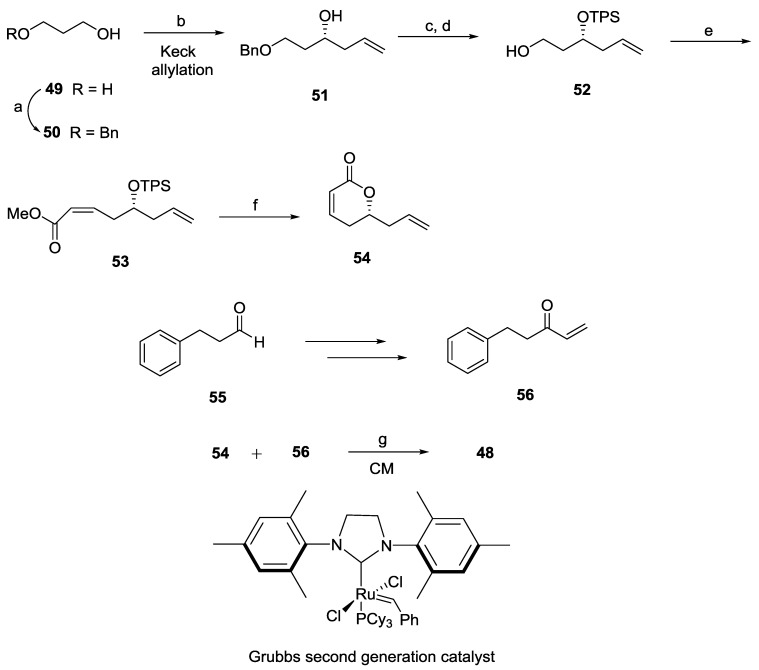
Reagents and conditions: (a) BnBr, NaH, TBAI, THF, 0 °C to r.t., 2 h, 85%; (b) (i) IBX, dry DMSO, CH_2_Cl_2_, 5 h, 88%; (ii) (*R*)-BINOL, 4 Å MS, Ti(*^i^*PrO)_4_, allyltributylstannane, CH_2_Cl_2_, –78 °C to –20 °C, 80%; (c) TBDPSCl, imidazole, CH_2_Cl_2_, 4 h, 95%; (d) Li in naphthalene, −20 °C, 3 h, 81%; e) (i) IBX, dry DMSO, CH_2_Cl_2_, 5 h, 85%; (ii) MeO_2_CCH_2_P(O)(OCH_2_CF_3_)_2_, NaH, THF, −78 °C, 2 h, 76%; (f) 3% HCl in MeOH 30 min, 78%; (g) Grubbs second-generation catalyst (5 mol %), CH_2_Cl_2_, 40 °C, 12 h, 74%.

The first stereoselective total synthesis of rugulactone (**48**) was reported by Venkateshwarlu et al. [38] as well as by Yadav et al. [39]. The first group used 1,3-propane diol (**49**) as the starting material (Scheme 10). It was converted to monobenzylether (**50**), which was oxidized with IBX and the resulting aldehyde underwent Keck allylation to form the homoallylic alcohol **51** (*ee* 97.5%). Protection of the hydroxyl group and removal of the benzyl group compound **51** yielded the alcohol **52**. The latter was oxidized with IBX, and the corresponding aldehyde was converted to the unsaturated ester **53** (*Z*:*E* = 95:5) by Still–Gennari modification of the Horner–Emmons olefination reaction. Treatment with 3% HCl in MeOH **53** yielded the pyranone **54**.

The ether part of rugulactone, fragment **56** was prepared from phenyl propanol (**55**) by treatment with vinyl magnesium bromide followed by oxidation of the generated alcohol with IBX. Finally, the cross-metathesis reaction of **54** and **56** using a second-generation Grubbs catalyst produced the natural rugulactone (**48**).

**Scheme 11:**
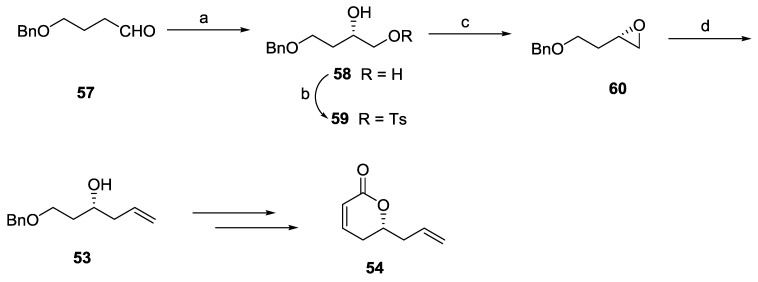
Reagents and conditions: (a) i) PhNO, D-proline (25 mol %), CH_3_CN, −20 °C, 24 h; then, MeOH, NaBH_4_; ii) CuSO_4_ (30 mol %), MeOH, 0 °C, 10 h, 85% (over two steps); (b) TsCl, Bu_2_SnO, Et_3_N, 0 °C to rt, 4 h, 81%; (c) K_2_CO_3_, MeOH, rt, 1 h, 93%; (d) Vinyl magnesium bromide, THF, CuI, −20 °C, 1 h, 88%.

Venkateshwarlu et al. in a later publication [41] showed the introduction of chirality through D-proline catalyzed α-aminoxylation of the aldehyde **57** (Scheme 11).

**Scheme 12:**
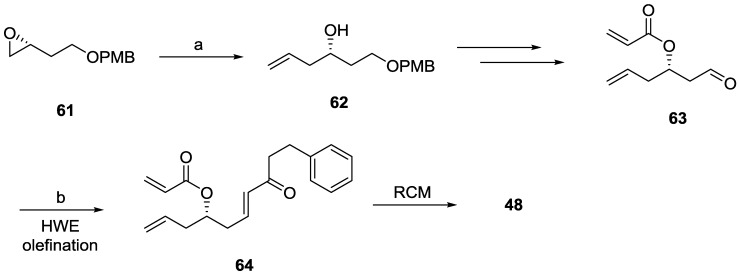
Reagents and conditions: (a) Vinylmagnesium bromide, CuI, THF, 0 °C, 2 h, 91%; (b) NaHMDS, dimethyl (2-oxo-4-phenylbutyl) phosphonate, 0 °C, 12 h, 87%.

Yadav et al. [39] initiated their synthesis from the chiral epoxide **61** (Scheme 12), which was prepared from the known corresponding racemic compound by Jacobsen’s hydrolytic kinetic resolution using (*R*,*R*)-(Salen) Co^III^ (OAc) catalyst. Epoxide **61** was reacted with vinyl magnesium bromide and CuI to form the homoallyl alcohol **62** (*ee* 87%). The esterification of **62** with acryloyl chloride, removal of the hydroxyl protection, and subsequently oxidation with DMP yielded the aldehyde **63**. This aldehyde **63** was subjected to Horner–Wadsworth–Emmons homologation using dimethyl (2-oxo-4-phenylbutyl) phosphonate to furnish the unsaturated ketone **64**. Finally, by treatment of this ketone (**64**) with Grubb’s first-generation catalyst, rugulactone (**48**) was formed.

**Scheme 13:**
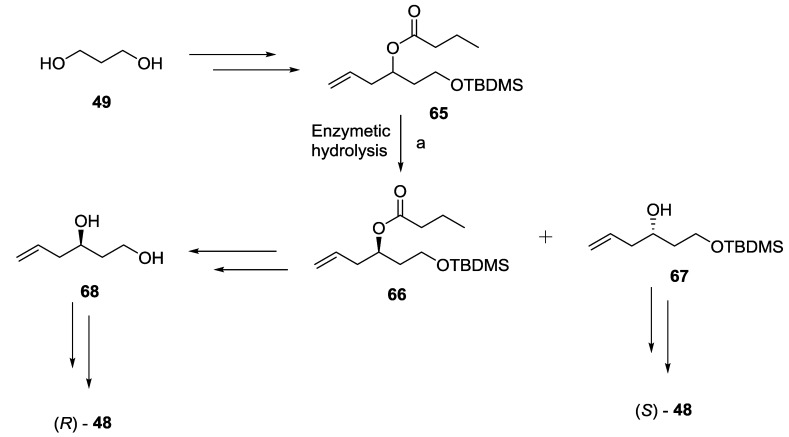
Reagents and conditions: (a) Tris–HCl buffer, 0.05 M, pH 7.5; *Candida rugosa lipase*, 48 h.

In a chemoenzymatic synthetic approach, both (*R*)- and (*S*)-rugulactone were prepared by applying the *Candida rugosa* lipase to hydrolyze the butyrate ester of the protected 3-hydroxy homoallylic alcohol **65** (Scheme 13) [42]. The key intermediates (*R*)-**66** (*ee* > 99%) and (*S*)-**67** (*ee* > 98%) were obtained with high enantiomeric purity. The ester **66** was hydrolyzed and deprotected to form (*R*)-**68**. Both the alcohols (*R*)-**68** and (*S*)-**67** were converted to (*R*)-**48** and (*S*)-**48** respectively following the earlier established method (Scheme 10). (*R*)-**48** is the naturally occurring rugulactone.

In another chemoenzymatic synthesis of rugulactone (**48**), chirality was induced by a stereoselective enzymatic reduction of a ketoester employing NADPH-dependent ketoreductase [40].

**Scheme 14:**
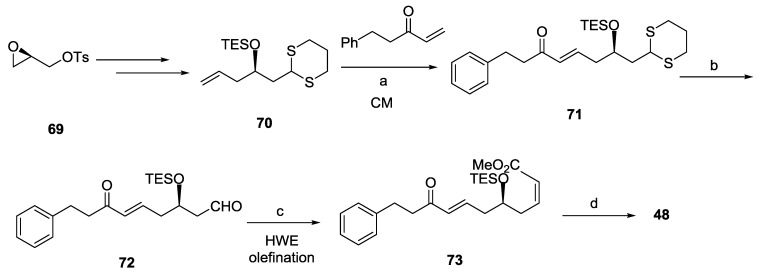
Reagents and conditions: (a) Grubbs second-generation catalyst (5 mol %), CH_2_Cl_2_, 40 °C, 12 h, 72%; (b) CaCO_3_, MeI, MeCN-H_2_O (9:1), 45 °C to r.t., 2.5 h; (c) MeO_2_CCH_2_P(O)(OCH_2_CF_3_)_2_, KHMDS, 18-crown-6, THF, −78 °C, 52%; (d) 80% AcOH, 60 °C, 24 h, 86%.

A chiral-pool method [43] was developed by Allais et al. for the asymmetric synthesis of rugulactone (**48**) (Scheme 14). The starting material was commercially available (2*S*)-glycidyl tosylate (**69**), which was converted to the olefin **70**. The olefin **70** was subjected to a cross-metathesis reaction with 5-phenyl-pent-1-en-3-one using Grubb’s II catalyst to furnish the α,β-unsaturated ketone **71**. The thioacetal group of **71** was removed, and the generated aldehyde **72** underwent a Still–Gennari olefination with methyl P,P-bis (2,2,2-trifluoromethyl) phosphonium acetate to form the unsaturated ester **73**. The latter on treatment with AcOH yielded natural rugulactone (**48**).

**Scheme 15:**
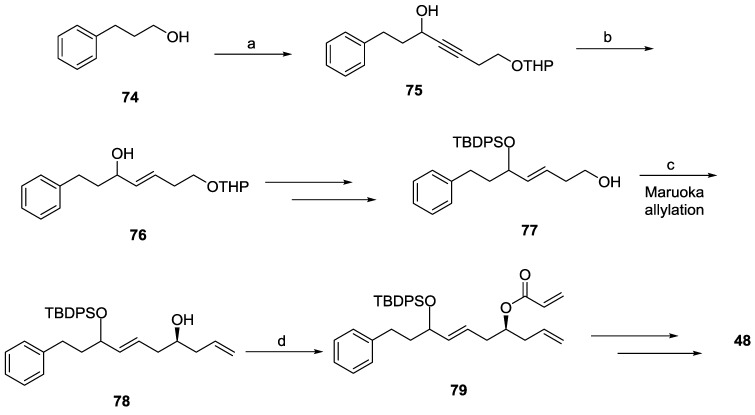
Reagents and conditions: (a) i) Oxalyl chloride, CH_2_Cl_2,_ DMSO, Et_3_N, −78 °C, 2.5 h, 82%; ii) BuLi, dry THF, THP-protected homopropargyl alcohol, −78 °C, 3 h; 87%; (b) LiAlH_4_, dry THF, 0 °C to reflux, 3 h; 78%; (c) i) IBX, DMSO, CH_2_Cl_2_, 0 °C to r.t., 2 h; 76%; ii) (*S*,*S*)-**I**, allyl(tributyl)tin, −15 °C to 0 °C, 16 h, 74%; (d) Acryloyl chloride, Et_3_N, DMAP, CH_2_Cl_2_, 0 °C to r.t., 4 h, 78%.

Das et al. achieved the total synthesis of rugulactone (**48**) using 3-phenyl propanol (**74**) as the starting material and applying Maruoka allylation and ring-closing metathesis as the key steps (Scheme 15) [44].

Compound **75** was prepared from **74** by oxidation of the latter under Swern conditions and treatment of the corresponding aldehyde with THP protected homopropargyl alcohol. The alkenol **76** was obtained by reduction of **75** with LiAlH_4_ and subsequently, it was converted to **77**. This alcohol (**77**) was oxidized with IBX, and the resulting aldehyde was subjected to Maruoka allylation [49] to form the homoallylic alcohol **78** (*ee* 97%). The latter was esterified with acryloyl chloride and the ester **79** was then converted to rugulactone (**48**).

**Scheme 16:**
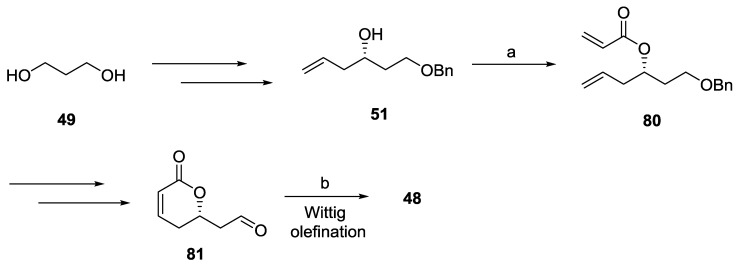
Reagents and conditions: (a) prop-2-enoyl chloride, CH_2_Cl_2_, Et_3_N, DMAP, 0 °C to r.t., 30 min, 96%; (b) PhCH_2_CH_2_COCH=PPh_3_, C_6_H_6_, reflux, 14 h, 72%.

Das et al. also synthesized rugulactone (**48**) through an alternative route (Scheme 16) [47]. They prepared the chiral aldehyde **81** from propane 1,3-diol applying Maruoka allylation and ring-closing metathesis. This aldehyde **81** underwent Wittig olefination with the phosphorane, PhCH_2_CH_2_COCH=PPh_3_ to yield rugulactone (**48**).

**Scheme 17:**
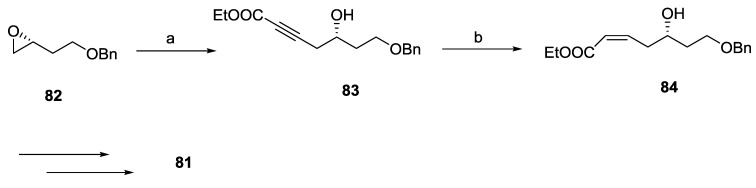
Reagents and conditions: (a) Ethyl propionate, BF_3_.Et_2_O, *n*-BuLi, –78 °C, THF, 93%; (b) H_2_, Lindlar’s catalyst, quinoline, EtOAc, 91%.

**Scheme 18:**
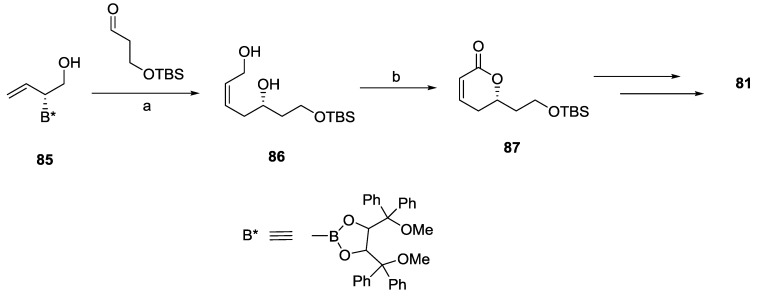
Reagents and conditions: (a) CH_2_Cl_2_, 0 °C to r.t., 98.4% *ee*, 92%; (b) PhI(OAc)_2_, TEMPO, CH_2_Cl_2_, r.t., 4 h, 92%.

The intermediate **81** was prepared by Barua et al. [45] from the chiral epoxide **82** (Scheme 17) and by Pietruszka et al. [46] from the allylic boronic ester **85** (Scheme 18).

In a synthetic approach, Reddy and Singh [48] applied Sharpless asymmetric epoxidation of an allyl alcohol to generate a chiral alcohol, which was applied as a key intermediate. The total synthesis of racemic rugulactone has also recently been reported [50].

### 2.3. Synargentolide A


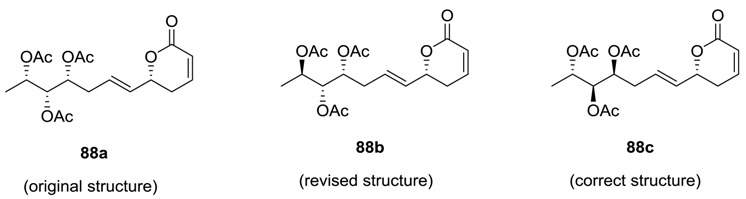


**Scheme 19:**
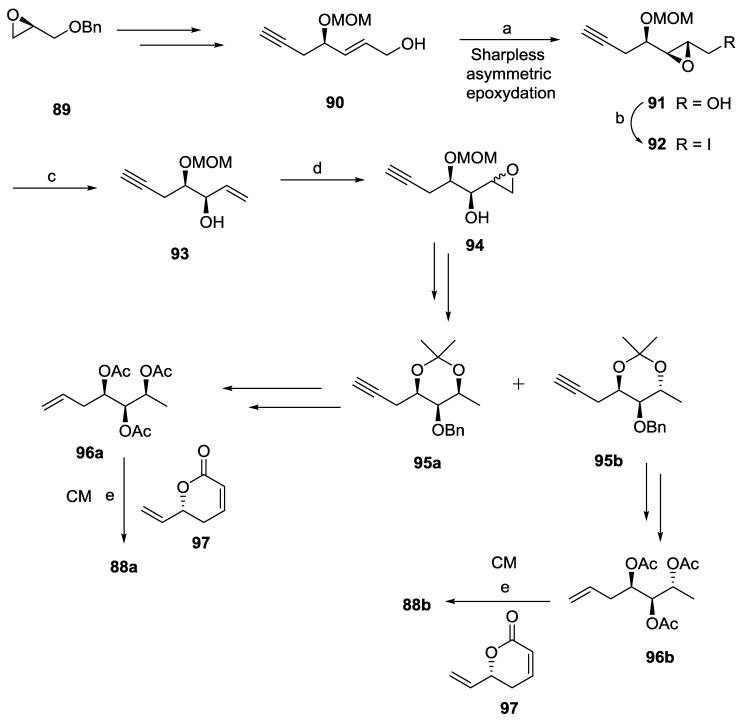
Reagents and conditions: (a) l-(+) DIPT, Ti(*^i^*PrO)_4_, cumenehydroperoxide, CH_2_Cl_2_, −24 °C, 4 h, 98%; (b) TPP, I_2_, imidazole, Et_2_O/CH_3_CN (3:1), 0 °C to rt, 20 min, 92%; (c) Zn dust, EtOH, reflux, 2 h, 92%; (d) i) m-CPBA, NaHCO_3_, CH_2_Cl_2_, −10 °C, 10 h, 92%, *dr* (1:1); ii) BnBr, NaH, THF, 0 °C to rt, 3 h, 95%; (e) Grubbs second-generation catalyst, refluxing CH_2_Cl_2_, 2 h, 65%.

Synargentolide A was isolated from the South African plant, *Syncolostemon argenteus* [51]. The compound is a member of 6-substituted α, β-unsaturated δ-lactones, which are well known for their cytotoxic and antitumor properties [3,6]. The structure of the compound was originally proposed as **88a** on the basis of spectroscopic studies, Mosher ester analysis, and acetonide formation [51]. Macro et al. synthesized the structure **88a** and observed that the synthetic compound was not identical to natural product [52]. Sabitha et al. also synthesized **88a** and its one stereoisomer **88b** (Scheme 19) [53]. Their synthesis was initiated with the known (*R*)-benzyl glycidyl ether **89**, which was converted to allyl alcohol **90**. The latter was subjected to Sharpless asymmetric epoxidation to form the single isomer **91**. This epoxide (**91**) was transformed to the alcohol **93**, which on epoxidation generated the epoxide **94**. Later, this epoxide (**94**) was converted to acetonides **95a** and **95b**.

Next, these acetonides **95a** and **95b** furnished separately the triacetates **96a** and **96b** respectively by deprotection, acetylation, and partial reduction. Finally, the cross-metathesis reaction between **95a**/**95b** and vinyl lactone (**97**) using Grubbs second-generation catalyst produced **88a**/**88b**. After inspection of the NMR spectra of **88a** and **88b**, the authors revised the structure of synargentolide A as **88b**.

**Scheme 20:**
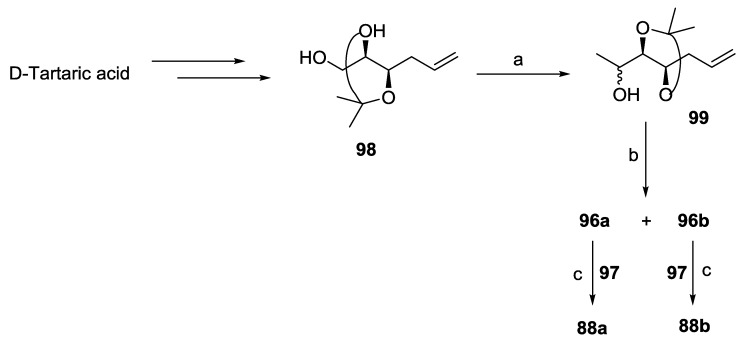
Reagents and conditions: (a) i) (COCl)_2_, DMSO, Et_3_N, CH_2_Cl_2_, 1 h, 89%; ii) MeMgBr, Et_2_O, −50 °C, 2 h, 62%; (b) i) MeOH, 2N HCl, r.t., 1 h, ii) Ac_2_O, Et_3_N, DMAP, CH_2_Cl_2_, 0 °C to r.t., 1 h, 92% (2 steps); (c*)* Grubbs second-generation catalyst, refluxing CH_2_Cl_2_, 2 h, 67% for **88a** and 66% for **88b**.

Several other syntheses of **88b** have recently been reported [54,55,56,57,58,59]. Das et al. developed an efficient synthesis of both **88a** and **88b** starting from D-tartaric acid (Scheme 20) [56]. The compound was converted to the alcohol **98**, which was subjected to Swern oxidation, and the corresponding aldehyde was treated with methyl magnesium bromide to produce the second alcohol **99**. The deprotection and acetylation of this compound (**99**) yields **96a** and **96b**, which were then converted to **88a** and **88b**, respectively (following the method shown earlier in Scheme 19).

It is interesting to mention here that the calculation of density functional theory (DFT) NMR parameters has recently suggested that both the structures **88a** and **88b** are incorrect and **88c** is the correct structure of the natural synargentolide A [60].

### 2.4. Synargentolide B


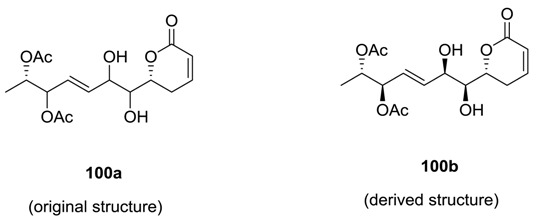


**Scheme 21:**
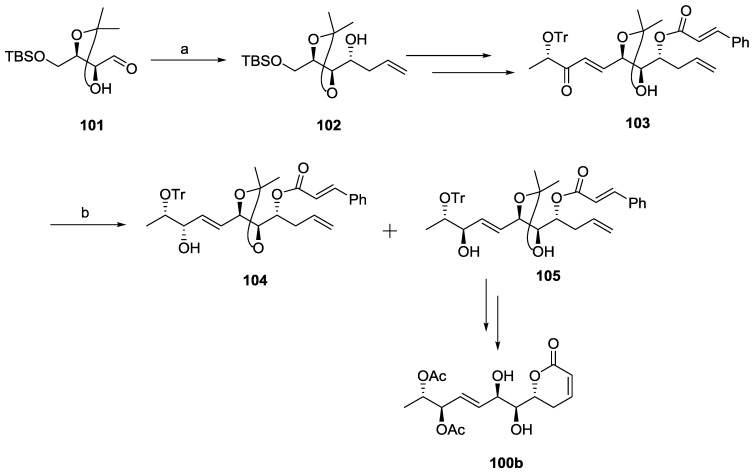
Reagents and conditions: (a) allylmagnesiumbromide, THF, 0 °C, 1 h, 40%; (b) NaBH_4_/CeCl_3_, MeOH, −78 °C, 1 h, 94%.

Synargentolide B was isolated from the South Africa species *Syncolostemon argenteus* [51]. Its structure was tentatively proposed as **100a**. Prasad and Gutala [61] carried out the total synthesis of possible diastereoisomers of the compound (Scheme 21) and derived the structure of the natural product as **100b**, which was earlier reported for a constituent of *Hyptis oblongifolia* [62].

Compound **100b** was synthesized [61] from the aldehyde **101**, which was converted to the major allyl alcohol **102**. This allyl alcohol **102** was elaborated to the ester **103**. On the reduction of **103** with NaBH_4_/CeCl_3_, two diastereoisomeric compounds **104** and **105** were obtained. Compound **104** was transformed to **105** by Mitsunobu inversion. Subsequently, **105** was converted to synargentolide B (**100b**) by reaction sequences involving deprotection, acetylation, and cross-metathesis.

**Scheme 22:**
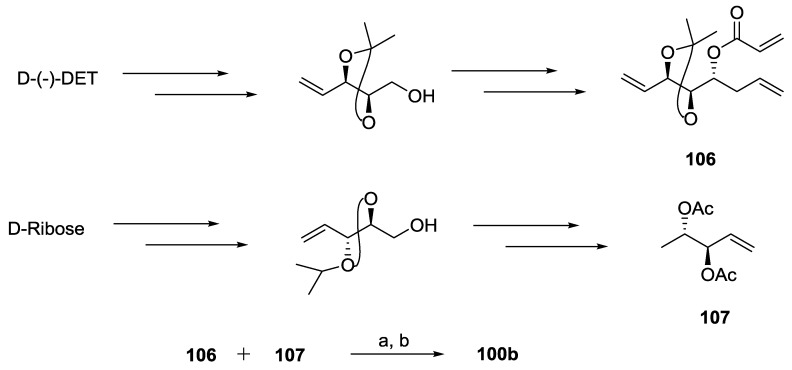
Reagents and conditions: (a) Grubbs second-generation catalyst, benzene, r.t. to reflux, 1.5 h, 83%; (b) TFA, CH_2_Cl_2_, 12 h, 87%.

In a tandem ring-closing/cross-metathesis approach for the synthesis of synargentolide B (**100b**), d-(-)-diethyl tartarate and d-ribose were used as starting materials (Scheme 22) [63].

**Scheme 23:**
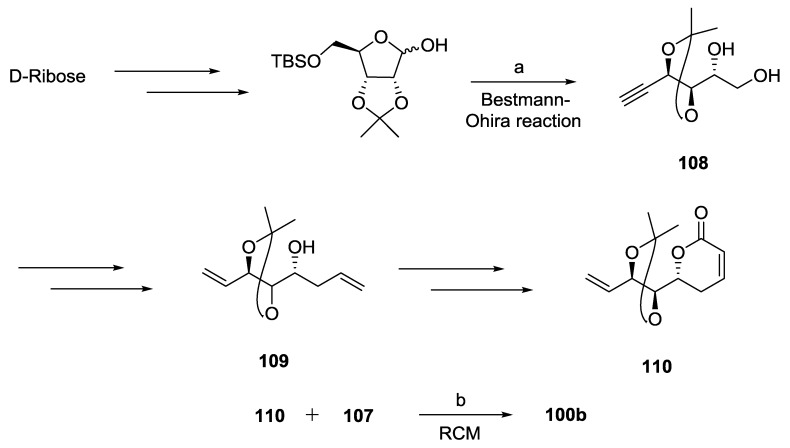
Reagents and conditions: (a) Bestmaan–Ohira reaction, reflux, 8 h, 65%; (b) i) Grubbs second-generation catalyst, CH_2_Cl_2_, reflux, 6 h, 67%; ii) PTSA, MeOH, reflux, 12 h, 78%.

Akkewar et al. obtained the diacetyl compound **107** from l-ascorbic acid, and they prepared the other part of **100b** from d-ribose employing the Bestmann–Ohira reaction, zinc allylation, and ring-closing metathesis (Scheme 23) [64].

Liu et al. prepared the intermediates **107** and **110** from L-ethyl lactate and D-mannitol respectively [65]. They also synthesized the enantiomer of natural synargentolide B [66]. A diastereoselective synthesis of 5′-*epi*- synargentolide B has also been reported [67].

**Scheme 24:**
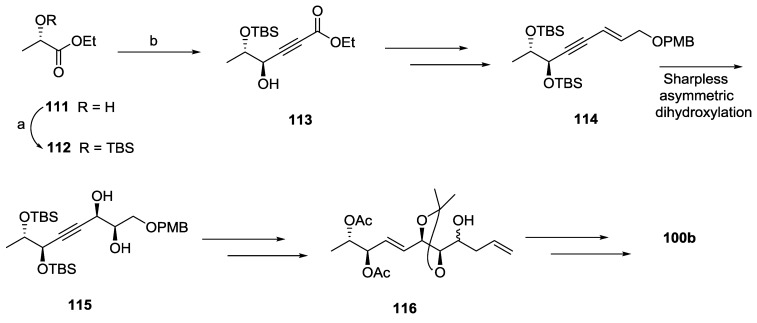
Reagents and conditions: (a) TBSCl, CH_2_Cl_2_, imidazole, r.t., 8 h, 93%; (b) (i) DIBAL-H, CH_2_Cl_2_, −78 °C, 0.5 h, 84%; (ii) ethyl propiolate, LIHMDS, THF, −78 °C to r.t., 3 h, 76%.

Suresh Babu et al. followed a different strategy for the stereoselective synthesis of synargentolide B (Scheme 24) [68]. They started their synthesis with ethyl (*S*)-2-hydroxypropanoate (**111**), which was protected to form **112**. The latter on reduction with DIBAL-H followed by treatment with ethyl propiolate and LiHMDS furnished the hydroxyl ester **113**. This ester (**113**) was subsequently converted to the protected allyl alcohol **114** following a reaction sequence involving protection, reduction, and Wittig olefination. Compound **114** underwent Sharpless asymmetric dihydroxylation using an AD-mix-β to form the diol **115** (*dr* 97.5:2.5). Next, this diol (**115**) was used to produce the allyl alcohol **116**, which was converted to two isomeric acryloylesters, and the major isomer was subsequently transformed to natural synargentolide B (**100b**).

### 2.5. Synrotolide


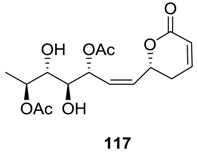


Synrotolide (**117**) was isolated from the leaves of *Syncolostemon ratundifolius* [69]. Its structure was determined from spectroscopic analysis and X-ray crystallographic studies. The structure of synrotolide (**117**) is interesting, as it contains a five-chiral center and a *cis*-double bond. Initially, the synthesis of its diacetate was reported [70,71], and later, the total synthesis of the natural product was published [72,73].

**Scheme 25:**
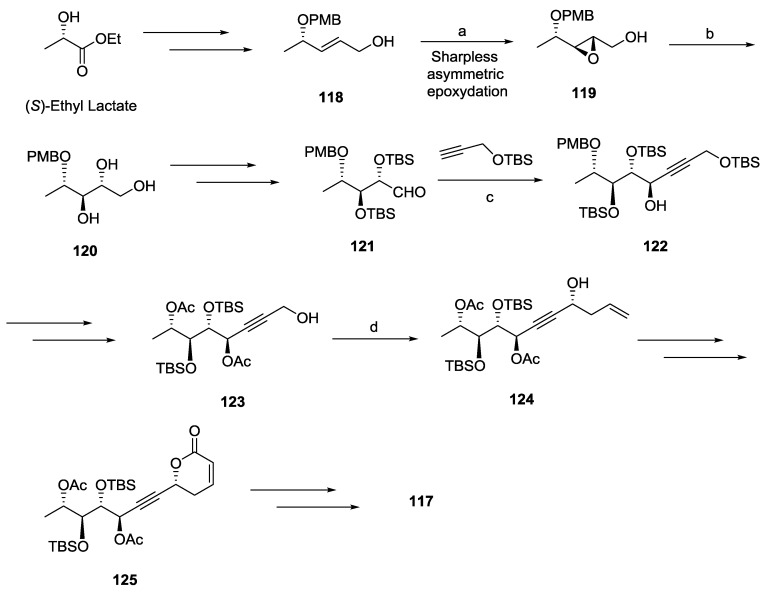
Reagents and conditions: (a) Ti(*^i^*PrO)_4_, (+)-DIPT, TBHP, CH_2_Cl_2_, −20 °C, 24 h, 91%; (b) 0.5 N NaOH, *t*-BuOH:H_2_O (1:5), 75 °C, 15 h, 72%; (c) *n*-BuLi, THF, −78 °C, 2 h, 91%; (d) i) IBX, DMSO, CH_2_Cl_2_, 0 °C to r.t., 2 h; ii) (+)IPC_2_Ballyl, Et_2_O, −100 °C, 81%, over two steps.

The total synthesis of synrotolide (**117**) was started from (*S*)-ethyl lactate, which was converted to the allyl alcohol **118** (Scheme 25) [72]. The Sharpless epoxidation of this allyl alcohol (**118**) using l-(+)-DIPT and TBHP yielded the epoxy alcohol **119** (*dr* 97:3). The ring opening of the epoxide **119** with 0.5 N NaOH in *t*-BuOH:H_2_O (1:5) afforded the alcohol **120**, which was converted to aldehyde **121**. Treatment of **121** with the protected hydroxyl propyne generated the compound **122** (*dr* 97:3), which by following protection/deprotection methodologies produced the alcohol **123**. Oxidation of the alcohol **123** with IBX and stereoselective allylation of the aldehyde with (+)-(IPC)_2_ Ballyl furnished the homoallyl alcohol **124** (*dr* 97:3). This homoallyl alcohol with different protecting groups was also prepared from (*S*)-ethyl lactate through an alternative route. Next, the alcohol **124** was converted to the pyranone derivative **125**, which on partial hydrogenation with Lindlar’s catalyst followed by treatment with H_2_SiF_6_ provided the natural synrotolide (**117**).

**Scheme 26:**
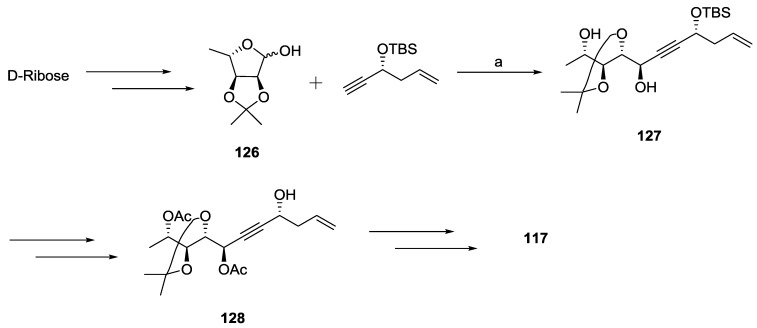
Reagents and conditions: (a) EtMgBr, THF, 0 °C to r.t., 3 h, 92%.

In another approach of the synthesis of synrotolide (**117**), the intermediates **128** (related to **124** with different protecting groups) was prepared from d-(-)-ribose (Scheme 26) [73]. This intermediate (**128**) was subsequently transformed to synrotolide (**117**).

The synthesis synrotolide (**117**) prepared by this method (Scheme 26) was evaluated for cyclotoxic activity. The compound was found to inhibit the growth of PANC1 cell lines [73].

### 2.6. Lippialactone


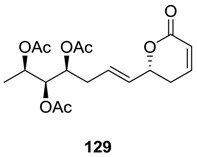


Lippialactone (**129**) was isolated from the acrial parts of *Lippia javanica* [74]. The compound is structurally related to synargentolide A (**88c**), but its stereoconfiguration is different. Lippialactone (**129**) was found to be active against the chloroquine-sensitive D10 strain of *Plasmodium falciparum*.

**Scheme 27:**
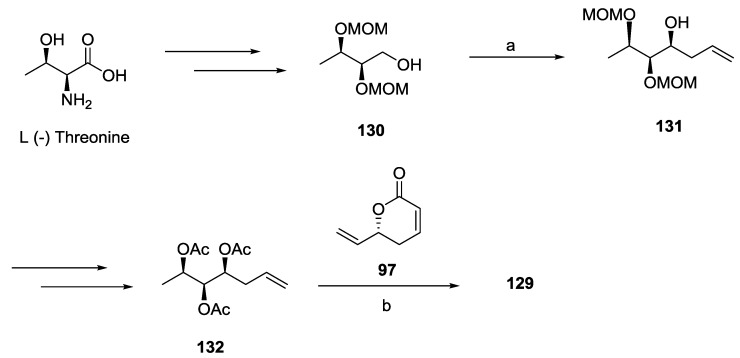
Reagents and conditions: (a) i) DMP, CH_2_Cl_2_, 0 °C to r.t., 1 h; ii) CH_2_=CHCH_2_SnBu_3_, MgBr_2_·OEt_2_, CH_2_Cl_2_, –78 °C, 2 h, 66% (over two steps); (b) Grubbs second-generation catalyst, CH_2_Cl_2_, reflux, 4 h, 68%.

The total synthesis of **129** was initiated from l (−)-threonine, which was converted to the alcohol **130** (Scheme 27) [75]. This alcohol (**130**) was oxidized, and the corresponding aldehyde was subjected to Keck allylation to produce the allyl alcohol **131** (*dr* = 84:16). Then, this allyl alcohol (**131**) was transformed to the required triacetate **132**. Finally, the cross-metathesis reaction between the triacetate **132** and vinyl lactone (**97**) using Grubbs second-generation catalyst furnished lippialactone (**129**).

**Scheme 28:**
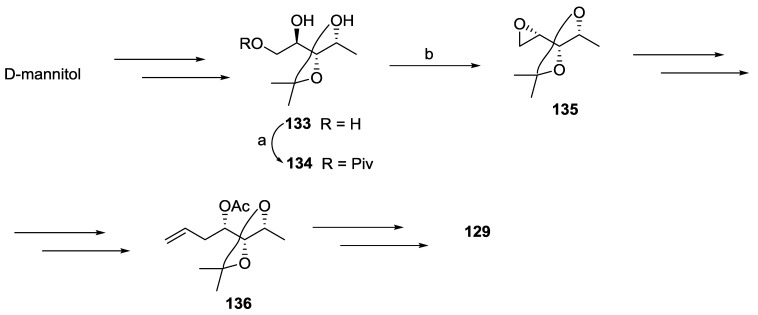
Reagents and conditions: (a) PivCl, Et_3_N, DMAP, CH_2_Cl_2_, 0 °C to r.t., 4 h, 87%; (b) i) MsCl, Et_3_N, DMAP, CH_2_Cl_2_, −80 °C to −20 °C, 12 h, ii) K_2_CO_3_, MeOH, rt, 2 h, 85% for two steps.

In another total synthesis of lippialactone (**129**), D-mannitol was used as the starting material (Scheme 28) [76]. It was converted to the diol (**133**), which was esterified with PivCl to form the ester **134**. Mesylation of the ester **134** and then treatment with anhydrous K_2_CO_3_ yielded the epoxide **135**. Ring opening of the epoxide (**135**) with vinyl magnesium bromide and CuI furnished a homoallyl alcohol, which on acetylation afforded the monoacetate **136**. The latter was converted to lippialactone (**129**) by a cross-metathesis reaction with vinyl lactone (**97**) followed by deprotection and acetylation.

### 2.7. Spicigerolide


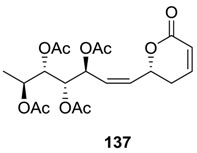


Spicigerolide (**137**) was isolated from the Mexican traditional medicinal plant, *Hyptis spicigera* [5]. The compound showed cytotoxic activity is some cell tumoral lines. The first synthesis of the compound [77,78] was described by Marco et al. [12]. Later, some other total syntheses of **137** were reported [79,80].

**Scheme 29:**
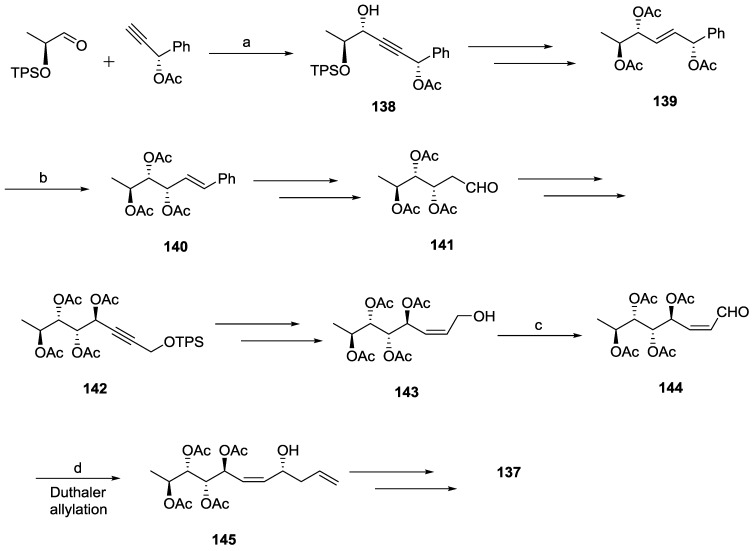
Reagents and conditions: (a) Zn(OTf)_2_, Et_3_N, (-)-NME, toluene, 4 h, 95%; (b) PdCl_2_(NCPh)_2_, CH_2_Cl_2_, r.t., 24 h, 70%; (c) Swern oxidation conditions; (d) CpTiCl-(*S*,*S*)-TADDOL, Et_2_O, CH_2_=CHCH_2_MgBr, –78 °C, 2.5 h, 84%.

Garcia et al. used protected (*S*)-lactaldehyde as the starting material (Scheme 29) [79]. This was treated with (*R*)-1-phenylprop-2-ynyl acetate (**137**) under Carreira’s condition [81] to form the *anti*-*syn* alcohol **138**, which was converted to the olefin **139**. This olefin (**139**) was subjected to a Pd-catalyzed [3,3]-sigmatropic rearrangement to form the triacetate **140**. The latter was transformed to the aldehyde **141**, which on treatment with 2-*tert*-butyldiphenylsilyloxy-1-propyne followed by acetylation afforded the tetraacetyl compound **142** as a single diastereoisomer. The partial hydrogenation of **142** using Lindlars’ catalyst and removal of the silicon-protecting group furnished the allyl alcohol **143**. Next, the later was oxidized to the aldehyde **143** under Swern condition, and this aldehyde (**144**) was allylated using Duthaler’s Ti-TADDOL-mediated allylation [82] to form the alcohol **145** (*dr* 87:13). Compound **145** with proper stereoconfiguration was subsequently converted to spicigerolide (**137**).

**Scheme 30:**
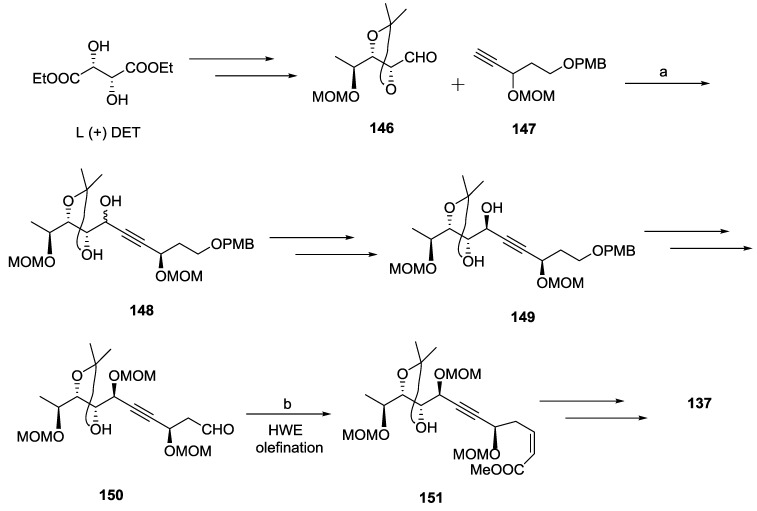
Reagents and conditions: (a) EtMgBr, THF, reflux, 2 h, 80%, (1:1 *dr*); (b) NaH, (F_3_CCH_2_O)_2_POCH_2_COOCH_3_, THF, −78 °C, 1 h, 80%.

In a recent synthetic approach, spicigerolide (**137**) was prepared from l-(+)-DET, which was transformed to the aldehyde (**146**) (Scheme 30) [80]. The Grignard reagent prepared from this aldehyde and ethyl bromide and Mg was treated with the alkyne **147** to produce the alcohol **148** (1;1 mixture of diastereoisomers). The alcohol (**148**) was oxidized, and the corresponding ketone was reduced using (*S*)-CBS catalyst [83] to furnish the chiral propargyl alcohol **149** (*dr* 9:1). Next, the aldehyde **150** generated from this alcohol (**149**) was used for chain elongation using a Still–Gennari reagent to form the ester **151**, which was subsequently converted to spicigerolide (**137**).

Recently, a total synthesis of an epimer of spicigerolide has also been reported. d-xylose was employed as a chiral source to generate the four stereogenic centers in the side chain [84].

### 2.8. Cryptofolione


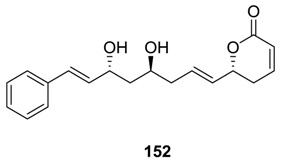


Cryptofolione (**152**) was isolated from *Cryptocarya myrtrifolia* and *C. moschata*, which are indigenous to South Africa and Brazil, respectively [85,86]. The compound was evaluated to be active against the trypomastigots of *Trypanosoma eruzi*, reducing their number by 77% at 250 µg/mL. The first synthesis of **152** [87] was mentioned by Macro et al. [12]. In recent years, several other syntheses of the molecule have been reported [88,89,90,91,92].

**Scheme 31:**
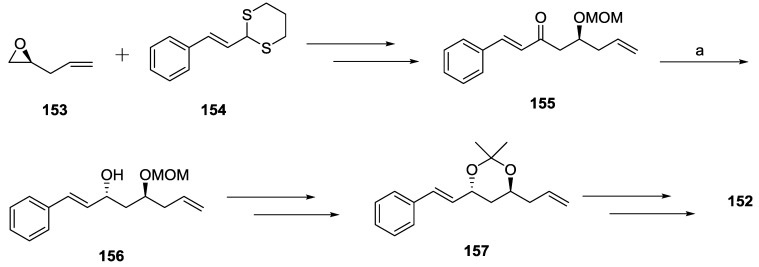
Reagents and conditions: (a) *S*-CBS catalyst, toluene, BH_3_.DMS, 0 °C, 0.5 h, 78%, 98% *de*.

In a recent synthetic approach, the required intermediate was prepared from a chiral allyl epoxide (**153**) (Scheme 31) [88]. This epoxide (**153**) was treated with acyl anion equivalent **154**, and the product was converted to the ketone **155**. A diastereoselective reaction of **155** with borane–dimethyl sulfide adduct using (*S*)-CBS catalyst yielded the chiral alcohol **156** (*de* > 95%). The removal of the MOM protecting group of the latter afforded the required diol-intermediate, which was transformed to the acetonide **157**. The same intermediate was also prepared by Prins cyclization of a chiral homoallylic alcohol with *trans*-cinnamaldehyde. Finally, the cross-metathesis reaction between **157** and the vinyl lactone (**97**) in the presence of Grubbs second-generation catalyst followed by treatment of the product with aq 4% HCl furnished the natural cryptofolione (**152**).

**Scheme 32:**
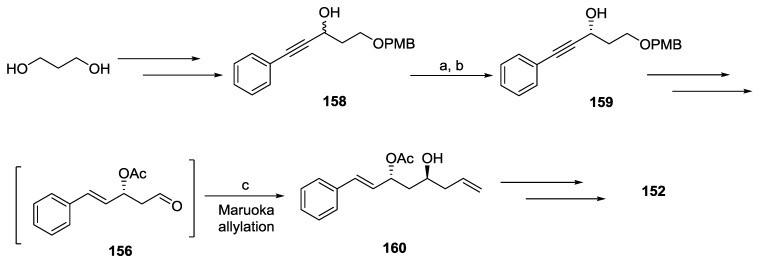
Reagents and conditions: (a) IBX, DMSO, CH_2_Cl_2_, 0 °C to r.t., 4 h, 88%; (b) (*R*)-(Me)-CBS (1.0 M in toluene), THF, BH_3_.Me_2_S, −20 °C, 2 h, 70%; (c) (*S*,*S*)-**I**, allyl tributyl stannane, CH_2_Cl_2_, 0 °C, 18 h, 74%,.

Das et al. initiated the synthesis of cryptofolione (**152**) starting from propane-1,3-diol, which was transformed to the alcohol **158** (Scheme 32) [89]. Oxidation of this alcohol (**158**) with IBX followed by reduction with BH_3_-Me_2_S using the catalyst (*R*)-2-methyl-CBS-oxazaborolidine furnished the chiral alcohol **159** (*ee* 97%). Acetylation of the free –OH group, removal of the PMB group, and oxidation of the generated alcohol with IBX formed an aldehyde. This aldehyde underwent Maruoka allylation to produce the allyl alcohol **160**, which was subsequently converted to cryptofolione (**152**).

In some current synthesis of natural cryptofolione, an asymmetric aldol reaction has been applied to generate the required chirality of the molecule [90,91,92]. Das et al. also completed the stereoselective synthesis of the non-lactonic portion of (*Z*)- cryptofolione [93].

### 2.9. Passifloricin A


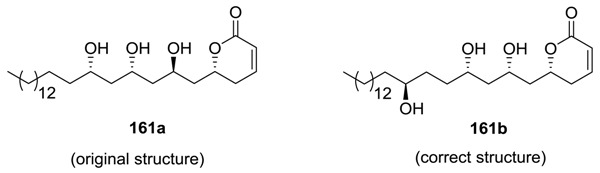


Passifloricin A was isolated from *Passiflora foctida var. hispida* [4]. Its structure was originally proposed to be **161a** from its spectroscopic data, and its correct structure was settled as **161b** on the basis of the syntheses of its different isomers [94,95,96,97]. The compound was found to inhibit impressive leishmanicidal and antiprotozoal properties [4].

**Scheme 33:**
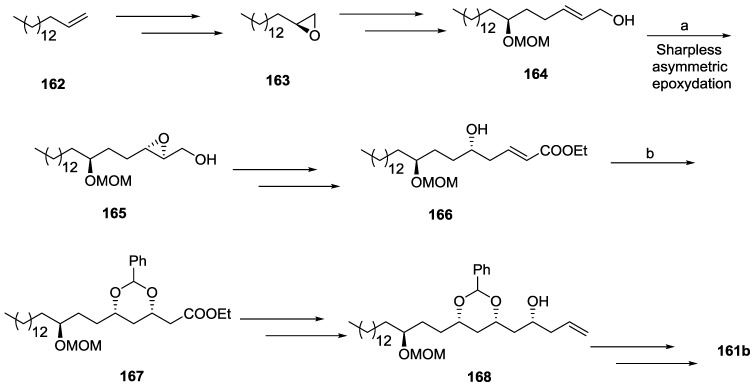
Reagents and conditions: (a) Ti(*^i^*PrO)_4_, (+)-DET, *^t^*BuOOH, CH_2_Cl_2_, 4 Å MS, −20 °C, 8 h, 85%; (b) PhCHO, KO*^t^*Bu, THF, 0 °C, 45 min, 72%.

In recent years, several new syntheses of passifloricin (**161b**) have been reported. Chandrasekar et al. prepared the compound starting from the olefin (**162**) (Scheme 33) [98]. This was converted to the chiral epoxide **163** by epoxidation followed by resolution of the racemic form using Jacobson’s catalyst. The epoxide **163** was transformed to the allyl alcohol **164** following a series of known reactions. This allyl alcohol **164** was subjected to Sharpless asymmetric epoxidation using (+)-DET to produce the chiral epoxy alcohol **165**. The latter was converted to the α,β-unsaturated ester **166**, which was treated with benzaldehyde and potassium *tert*-butoxide to form the acetal **167**. The ester group of **167** was reduced to aldehyde, and the product underwent Maruoka allylation to furnish the major syn-isomer **168**. This allyl alcohol (**168**) was subjected to its conversions to passifloricin A (**161b**) with proper stereo configuration.

A similar intermediate as **168** with different protecting groups was also prepared by a different research group [99]. They employed Prins cyclization [100] as the key step.

**Scheme 34:**
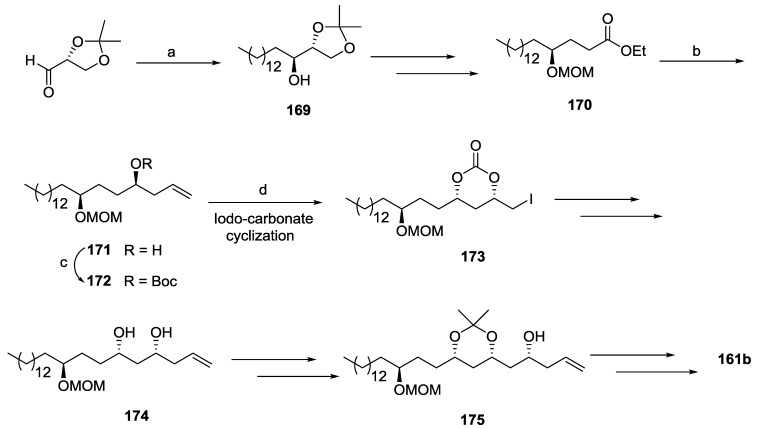
Reagents and conditions: (a) Me(CH_2_)_13_Br, Mg, THF, 70 °C, 8 h, 94%; (b) i) DIBAL-H, CH_2_Cl_2_, −78 °C, 2 h, 79%; ii) (*R*,*R*)-BINOL (10 mol%), allyl(tributyl)-stannane, 4 Å MS, CH_2_Cl_2_, 0 °C, 25 h, 81%; (c) (Boc)_2_O, CH_2_Cl_2_, Et_3_N, DMAP, 0 °C to r.t., 4 h, 93%; (d) *N*-Iodosuccinimide, MeCN, −20 °C, 2 h, 81%.

Das et al. accomplished the total synthesis of passifloricin A (**161b**) starting from protected glyceraldehyde and employing Maruoka allylation, iodo-carbonate cyclization, and olefin metathesis as the key reactions in their synthesis sequence (Scheme 34) [101].

Protected glyceraldehyde was treated with 1-bromotetradecane and the major product, *anti*-isomer **169**, was purified by chromatography. This compound (**169**) was converted to the ester **170**, which was reduced with DIBAL-H, and the resulting aldehyde was subjected to Maruoka allylation to give the allyl alcohol **171** (*ee* 97%). The free hydroxyl group of **171** was protected with Boc_2_O, and the product underwent iodo-carbonate cyclization with NIS to furnish the major syn-isomer **173** (>95%). The purified **173** was reacted with K_2_CO_3_ in MeOH, and the resulting epoxide was treated with vinyl Grignard reagent and CuI to produce the allyl 1,3-diol **174**. The protection of two hydroxyl groups of **174**, conversion of the olefin moiety to aldehyde, and again Maruoka allylation produced the required intermediate **175**, which generated passifloricin A in a stereoselective manner.

### 2.10. Strictifolione


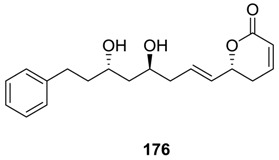


Strictifolione (**176**) was isolated from the stem bark of *Cryptocarya strictifolia* that grows in Indonesia [102]. Its structure was deduced from spectroscopic analysis. The compound was found to display antifungal property. Its earlier syntheses were mentioned by Macro et al. [12]. Recently, a large number of new syntheses of strictifolione (**176**) have been reported [92,103,104,105,106,107,108,109].

**Scheme 35:**
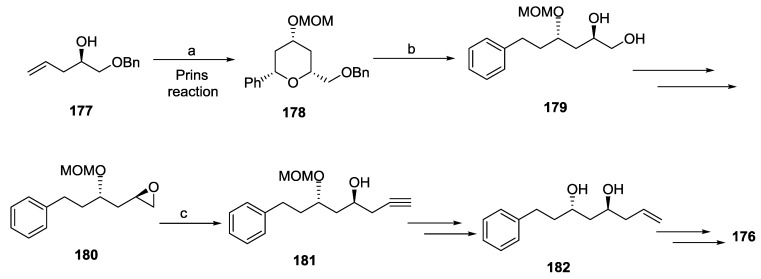
Reagents and conditions: (a) i) PhCHO, TFA, CH_2_Cl_2_, K_2_CO_3_, MeOH, r.t., 0.5 h, 59%; ii) MOMCl, Hunig’s base, 0 °C to r.t., 2 h, 90%; (b) Li-liq NH_3_, dry THF, −78 °C, 60%; (c) LiC≡CH, DMSO, 0 °C to r.t., 4 h, 80%.

In a recent synthesis, known chiral allyl alcohol **177** was used as a starting material (Scheme 35) [103]. The Prins reaction between **177** and benzaldehyde followed by hydrolysis of the generated trifluoroacetate and protection of the hydroxyl group afforded **178**. The tetrahydropyran ring of **178** was opened with Li in liquid NH_3_ to form the open chain compound **179**. The protection of the primary hydroxyl group of **179** as a tosyl derivative and treatment of the product with NaH yielded the epoxide **180**. The opening of this epoxide (**180**) with Li acetylide afforded the homopropargyl alcohol **181**. The partial reduction of **181** with Lindlar’s catalyst and deprotection of the MOM group furnished the required intermediate **182**. Finally, the cross-metathesis reaction between **182** and vinyl lactone **97** using Grubbs second-generation catalyst afforded natural strictifolione (**176**).

**Scheme 36:**
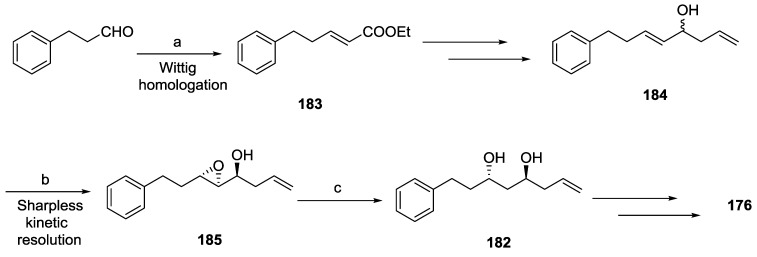
Reagents and conditions: (a) Ph_3_PCHCOOEt, CH_2_Cl_2_, r.t., 8 h, 84%; (b) Ti(*^i^*PrO)_4_, (+)-DIPT, TBHP, CH_2_Cl_2_, 4 Å MS, −20 °C, 5 h, 45%; (c) Red-Al, THF, 0 °C to r.t., 3 h, 77%.

During the studies [104,110,111,112,113,114] on the syntheses of natural pyranones, Das et al. accomplished the stereoselective total synthesis of strictifolione (Scheme 36) [104]. The starting material, phenyl propanal, was subjected to 2C-Wittig homologation with (carboethylmethylene) triphenylα phosphorane to produce the α,β-unsaturated ester **183**. Reduction of the ester **183** with DIBAL-H and allylation of the resulting aldehyde afforded the racemic alcohol **184**. The Sharpless kinetic resolution of **184** by applying (+)-DIPT yielded the chiral epoxy alcohol **185** (*ee* 97%). The epoxide ring of **185** was opened with Red-Al to give the intermediate **182**, which was transformed to strictifolione (**176**).

The same intermediate **182** or its protected form was also prepared by different other research groups by utilizing various synthetic methodologies, such as hydrolytic kinetic resolution [105,106], chemoenzymatic means [107], and asymmetric aldol reaction [92]. In addition, one modular approach that utilized phosphate tether mediate protocol was also developed for the synthesis of **182 [108]**.

**Scheme 37:**
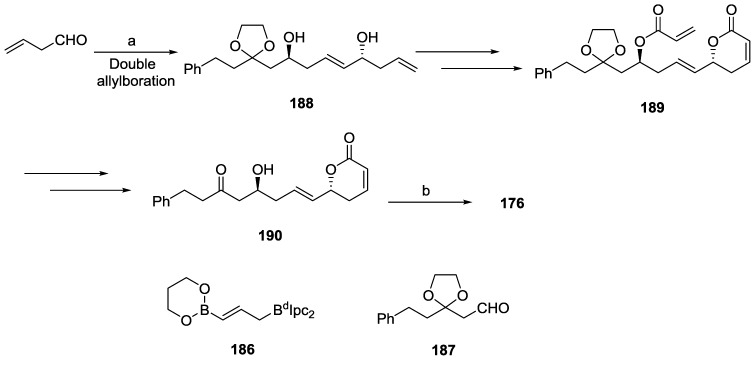
Reagents and conditions: (a) **186**, Et_2_O, −78 °C, 2 h, then **187**, r.t., 24 h, 55%, 92% *ee*; (b) Me_4_NBH(OAc)_3_, AcOH/CH_3_CN (1:1), −20 °C, 10 h, 91%.

An efficient synthesis of strictifolione (**176**) was achieved by She et al. by employing one-pot double allyl boration and ring-closing metathesis (Scheme 37) [109]. The required intermediate **188** was prepared by the treatment of 3-butenal with boryl-substituted allylborane **186** and then with the known aldehyde **187** utilizing a double allylboration methodology. Compound **188** was obtained with high diastereoselectivity and enanteioselectivity (*dr* ≥ 20:1, *ee* 92%). The esterification of this compound with acryloyl chloride and ring-closing metathesis of the resulting diester followed by deprotection of the product **189** furnished the ketone **190**. Finally, the reduction of this ketone (**190**) with Me_4_NBH (OAc)_3_ yielded strictifolione (**176**).

## 3. Conclusions

In the present article, we have described briefly the recent progress in the stereoselective total syntheses of natural pyranones having long-chain substituents. A large number of molecules have currently been synthesized by different workers following various synthetic approaches. As for examples, nine syntheses of dodoneine (from 2008) and 12 syntheses of rugulactone (from 2009) have been reported. The interesting structural features as well as promising biological activities of natural pyranones stimulated the research groups to develop new methodologies for their total syntheses. We have considered some important bioactive natural pyranones having long side chains and discussed the different modern approaches for their stereoselective syntheses. From this review, it is apparent that the rapid achievement in the diastereoselective and enantioselective synthetic protocols have made it possible to introduce proper chirality in the pyranone molecules. The ring-closing metathesis and cross-metathesis reactions have been largely utilized for the construction of their lactone rings and side chains, respectively. It is expected that the knowledge generated from the modern synthetic endeavors of the described natural pyranones in this article will enable the further development of more concise, efficient, and practical syntheses of this class of compounds.

## Abbreviations

HF	Hydrofluoric acid	TsCl	*Para*-toluene sulfonyl chloride
TBS	*tert*-Butyldimethylsilyl	TESCl	Triethylsilyl Chloride
DIBAL-H	Diisobutylaluminium hydride	DET	N,N-Diethyltryptamine
DIPEA	*N*,*N*-Diisopropylethylamine	(*S*)-CBS	(*S*)-5,5-Diphenyl-2-methyl-3,4-propano-1,3,2-oxazaborolidine
PTSA	*para*-toluenesulfonic acid	NIS	*N*-Bromo scuccinimide
PPTS	Pyridinium *p*-toluenesulfonate	TBSOTf	Trimethylsilyl trifluoromethanesulfonate
DIPT	Diisopropyltartrate	*t*-BuOK	Potassium *tert*-butoxide
TBHP	*tert*-Butylhydroperoxide	AcOH	Acetic acid
DMP	2,2-Dimethoxypropane	LiAlH_4_	Lithium aluminium hydride
TBSCl	*tert*-Butyldimethylsilyl chloride	Et_3_N	Triethyl amine
IBX	2-Iodoxybenzoic acid	*t*-BuOH	*tert*-Butyl alcohol
BINOL	1,1′-Bi-2-naphthol	NaBH_4_	Sodium borohydride
Ti(^i^PrO)_4_	Titanium isopropoxide	BnBr	Benzyl Bromide
PMB	*4*-*Methoxybenzyl*	THF	Tetrahydrofuran
KHMDS	Potassium hexamethyldisilazide	CHCl_3_	Chloroform
NF-kB	Nuclear factor kappa-light-chain-enhancer of activated B cells	DMAP	4-Dimethylaminopyridine
TBAI	*tert*-Butylammonium iodide	NaH	Sodium hydride
TBDPSCl	*tert*-Butyldiphenylsilyl chloride	NH_4_Cl	Ammonium chloride
NaHMDS	Sodium bis (trimethylsilyl) amide	NaIO_4_	Sodium periodate
NADPH	Nicotinamide adenine dinucleotide phosphate	Ac	Acetyl
THP	Tetrahydropyran	TFA	Trifluoroacetic acid
TEMPO	(2,2,6,6-Tetramethylpiperidin-1-yl)oxyl	MOMCl	Methoxymethyl chloride
TPP	Thiamine pyrophosphate		
